# Targeting inflammasome pathway by polyphenols as a strategy for pancreatitis, gastrointestinal and liver diseases management: an updated review

**DOI:** 10.3389/fnut.2023.1157572

**Published:** 2023-08-31

**Authors:** Abdelhafid Nani, Wafâa Tehami

**Affiliations:** Laboratory of Saharan Natural Resources, University of Ahmed Draia, Adrar, Algeria

**Keywords:** Western diet, NLRP3 inflammasome, polyphenols, pancreatitis, gastrointestinal and liver diseases, oxidative stress

## Abstract

Obesity, pancreatitis, cardiovascular, gastrointestinal (GI), and liver diseases have all been linked to the Western lifestyle, characterized by increased unhealthy food consumption and decreased physical activity. Besides obesity and pancreatitis, many GI and liver diseases are associated with inflammation. Inflammasomes are multi-protein complexes that mediate acute and restorative inflammatory pathways. However, many aberrations in inflammasome activity originate from shifts in dietary habits. Evidence reveals that dietary polyphenols effectively modulate inflammasome-associated dysfunctions. With a focus on pancreatitis, GI, and liver disorders, this review set out to provide the most relevant evidence for the therapeutic impact of polyphenols via the regulation of the inflammasome pathway. Overall, flavonoid and non-flavonoid polyphenols maintain intestinal eubiosis, downregulate NLRP3 inflammasome canonical pathway, and restore redox status via upregulating Nrf2/HO-1 signaling. These effects at the level of the intestine, the liver, and the pancreas are associated with decreased systemic levels of key pro-inflammatory cytokines, including TNF-α, IL-1β, and IL-6.

## 1. Introduction

Western (or more broadly “modern”) lifestyle, which is characterized by increased unhealthy food consumption and decreased physical activity, is linked to chronic diseases of civilization, including epithelial cell cancers, obesity, pancreatitis, cardiovascular, gastrointestinal (GI), and liver diseases (1–4). GI diseases encompass one or more of the four typical symptoms and signs: abdominal or chest pain, altered food ingestion, altered bowel movements, and GI tract bleeding (5). Many GI and liver diseases are associated with inflammation, which is a physiological response that can be triggered by invading microbes’ antigens or host inflammatory molecules (6, 7). Inflammasomes are multi-protein complexes that mediate acute and reparative inflammatory pathways (8). However, deregulated inflammasome activities can result in chronic inflammation underlying a series of pathologies, such as GI and liver disorders and pancreatitis (9–13). In addition to the gut microbiota, dietary habit changes are believed to underlie many host inflammatory responses (8, 14–16). Indeed, the release of free fatty acids (FFAs) in the human GI tract may trigger NLRP3 inflammasome-mediated inflammation (16–18). Therefore, a healthy diet rich in nutraceuticals can be an excellent strategy for managing GI and liver disorders, partly through regulating inflammasome activities (19, 20). Polyphenols are dietary nutraceuticals that have been reported to exhibit a number of biological activities, such as antioxidant, antimicrobial, anti-inflammatory, and anticancer properties (18). These bioactive substances are found in many cereals, vegetables, fruits, herbs, and beverages. Accumulating evidence reveals that polyphenols are effective in modulating inflammasome-associated dysfunctions, including pancreatitis, GI and liver disorders (21–24). Pellegrini et al. (25) recently provided an overview of NLRP3 inflammasome pathway modulation by secondary metabolites. Another recent review by Owona et al. (26) highlighted the role of flavonoids, the most abundant phenolic compounds, in modulating numerous inflammasome-associated chronic diseases. Therefore, studies have been expanding on polyphenols-induced inflammasome regulation as a prominent approach for managing and treating inflammation-associated diseases. This review aimed to provide up-to-date evidence for the therapeutic impact of polyphenols via the regulation of the NLRP3 inflammasome pathway, with a focus on pancreatitis, gastrointestinal and liver disorders.

## 2. Inflammasome activation by Western dietary patterns

Inflammasomes are intracellular multi-protein complexes made up of three components: a sensor molecule consisting of nucleotide-binding oligomerization domain (NOD)-like receptors (NLRs), an adaptor protein ASC (apoptotic speck-like protein containing), and an effector molecule procaspase-1 (12). NLR family pyrin domain-containing protein (NLRP) inflammasomes are innate immune sensors that are assembled following NLR’s recognition of multiple classes of damage-associated molecular patterns (DAMPs), in response to cell injury, and pathogen-associated molecular patterns (PAMPs) of invading microbes (8). Once assembled, NLRPs mediate the caspase-1 activation that results in proteolytic cleavage of pro-interleukin-1β (pro-IL-1β) and IL-18 into bioactive forms leading to a myriad of additional cytokines and chemokines upregulation or initiating pyroptotic cell death (pyroptosis) (27).

Approximately half of all deaths worldwide in the late 19th century was due to infectious diseases. This burden has dropped significantly in the last century, from 85 to 15%, thanks to significant developments such as widespread sanitation improvement. Contrarily, shifts in dietary habits, especially in Western societies, have contributed significantly to the emergence of non-communicable diseases. Furthermore, a Western-style calorie-rich diet is directly or indirectly responsible for over 80% of all deaths (28). Western diet (WD) patterns include a lot of high-glycemic/high-insulinemic carbohydrate foods like refined cereals, corn, potatoes, sugars (mainly sucrose and fructose), dairy products, and a considerable amount of fat (a fair amount of ω-6 polyunsaturated fatty acids “PUFAs”) and plenty of protein (29). Adherence to the Western dietary pattern enhances refined starchy carbohydrate amounts and free fatty acids (FFAs) release. All of which can result in sterile (or non-pathogenic) inflammation associated with alterations in gut microbiota (17, 22, 30–32). Indeed, a sedentary lifestyle and excessive intake of fat, starchy carbohydrates, and free sugars have been suggested to activate inflammasomes through uric acid, resulting in exacerbated oxidative stress and inflammation in the liver (31). It has also been shown that FFAs induce the expression of NLRP3 inflammasome complex-forming proteins in endothelial cells (17). Increasing evidence has established the association of WD with gut microbiota profile alterations and gut mucosal barrier disruption (32, 33). The gut mucosa disruption is a hallmark of inflammatory bowel diseases (IBD) characterized by an upregulated NLRP3 inflammasome pathway, which can be triggered following short chain fatty acids (SCFAs) binding to GPR43 of the enterocytes (19, 22, 32, 34). Moreover, a recent study showed that WD is associated with altered gene expression, resulting in an elevated risk for autoimmune pancreatitis (30). Since the liver is a key metabolic organ, WD patterns, including simple sugars, saturated fatty acids, trans fats, and animal proteins, play a crucial role in the onset and progression of liver pathologies such as steatosis and nonalcoholic fatty liver disease (NAFLD) (35). Indeed, high levels of alcohol absorption can cause hepatocyte death. Likewise, hepatocyte steatosis can be caused by a high energy intake of fat and sugar (31). All these factors can alter the gut microbiota composition, resulting in increased microbial translocation to the portal blood and increased PAMPs exposure in the liver. These PAMPs from the gut activate liver immune cells via PRRs, leading to increased IL-1β and IL-18 production through toll like receptor 4/nuclear factor-kappa B /NLRP3 (TLR4/NF-κB/NLRP3) inflammasome signaling pathway (36).

**Figure 1:**
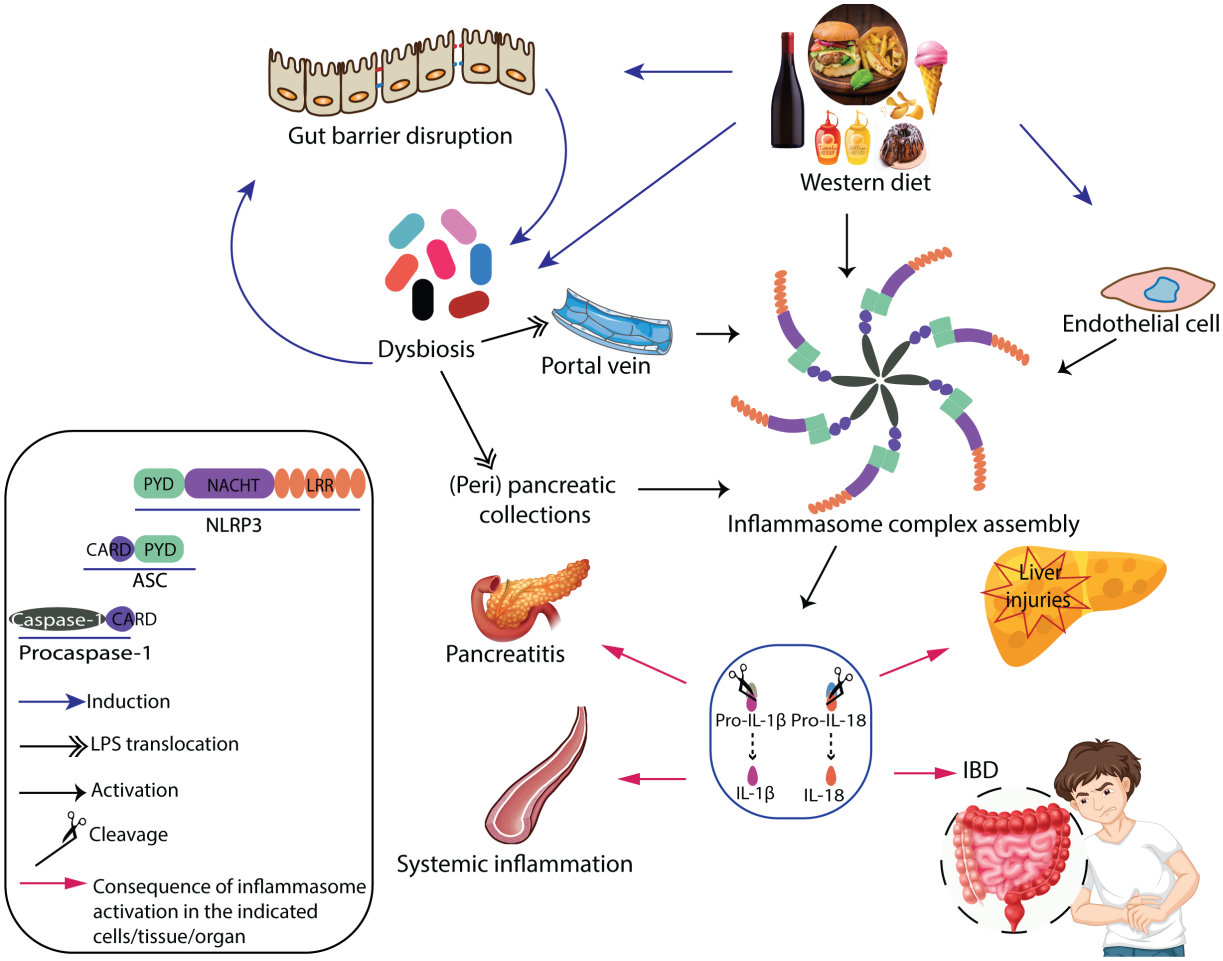
Graphical summary of the mechanisms underlying inflammasome complex-mediated inflammatory disorders by Western diet.

The main mechanisms underlying NLRP3 inflammasome complex-mediated inflammatory disorders by WD are overviewed in Figure 1.

## 3. Polyphenols impact on inflammasome-mediated diseases

The term polyphenols refers to a series of homologous compounds comprising a hydroxyl group bonded directly to a benzene ring. Thus, polyphenols are substances broadly distributed in the plant kingdom that contain multiple phenyl rings and at least one hydroxyl substituent (37). Their structure may vary from basic compounds like phenolic acids and stilbenes to more complex polymers with a high molecular weight, like tannins (38). Dietary polyphenols are frequently classified into flavonoid and non-flavonoid polyphenols. Plant-based polyphenols are potent anti-inflammatory agents, and this benefit may be mediated by their ability to modulate inflammasome activity (39). Indeed, *in silico* investigations showed that phenolics such as phenylpropanoids, curcumin, and epigallocatechin-3-gallate (EGCG) exhibited a high affinity with the NLRP3 inflammasome complex (40, 41). Thus, phenolic compounds may be potent inhibitors of NLRP3 inflammasome activation. Therefore, they can modulate NLRP3 inflammasome-associated inflammation and pathologies.

### 3.1. Pancreatitis

Acute pancreatitis (AP) is the most prevalent pancreatic pathology and the most likely cause of hospitalization among nonmalignant gastrointestinal illnesses (13). Evidence showed that AP may be linked to luminal lipids maldigestion, and the premature activation of the proteolytic proenzymes such as trypsinogen within the pancreatic acinar cells is expected in this pathology (42). It is demonstrated that the NLRP3 inflammasome is incriminated in pancreatitis onset and complications (13). Thus, inhibiting the NLRP3 inflammasome pathway might be an effective treatment for patients with severe pancreatitis. Phytochemicals have been reported to modulate inflammasome-mediated diseases (25, 26, 43). Accordingly, several studies investigated the impact of dietary polyphenols on inflammasome-mediated pancreatitis (21, 44–47). Aruna et al. (44) suggested that rutin, a flavonoid glycoside, curtails pancreatitis through the downregulation of ASC–NLRP3, resulting in reduced caspase-1 activation and decreased IL-1β, IL-18, and tumor necrosis factor-α (TNF-α) pro-inflammatory cytokines expression and production in alcohol and cerulein-induced pancreatitis model. It is well-established that systemic inflammation is common in severe AP (13). Interestingly, EGCG and other phenolic compounds have been shown to decrease systemic levels of key pro-inflammatory cytokines, including TNF-α, IL-1β, and IL-6 (21, 45, 47).

Reactive oxygen species (ROS) are the root cause of cellular oxidative stress, significantly contributing to AP pathogenesis (48). It is well known that endogenous and exogenous antioxidants counteract cellular ROS overproduction. Several phenolic compounds exhibited a preventive effect against AP severity and complications by mitigating mitochondrial ROS-triggered NLRP3 inflammasome activation or upregulating the nuclear factor erythroid 2-related factor 2 (Nrf-2) pathway (21, 44, 45, 49). Indeed, rutin reduced plasma levels of thiobarbituric acid reactive substances (TBARS), lipid peroxidation end by-products, and enhanced glutathione peroxidase (GPx), Superoxide dismutase (SOD), Catalase (CAT) activity in pancreatic tissue of alcohol and cerulein-induced pancreatitis model (44). Moreover, apocynin, a phenolic compound, decreased the ROS level in the lung of rats suffering from severe AP (45). Another study revealed that EGCG co-administration enhanced SOD activation and glutathione (GSH) level concomitant with decreased malondialdehyde (MDA) levels in the lung tissue of Balb/C mice suffering from AP induced with L-arginine (21).

### 3.2. Gastrointestinal pathologies

The gastrointestinal tract contains many immunocompetent cells and is the largest compartment for food digestion and nutrient absorption. The body’s most significant mucosal layer, the intestinal mucosa, is crucial to maintaining intestinal homeostasis. Nevertheless, nutrition, drugs, microbial stimuli, and exacerbated production of many metabolites normally produced in cells are risk factors for intestinal inflammation that underlies numerous intestinal disorders. Indeed, long-term exposure to inflammatory cytokines such as IL-1, IL-6, IL-8, TNF-α and interferon-gamma (IFN-γ) is linked to several illnesses, including IBD which include ulcerative colitis (UC) and Crohn’s disease (CD). These cytokines disrupt the homeostasis of the digestive system during an imbalanced inflammatory condition, which results in a chronic inflammatory process (50–52). Recent research has shown how crucial canonical NLRP3 inflammasome signaling is for controlling intestinal homeostasis and the risk for IBD (53).

Phenolic compounds have attracted the attention of several researchers due to their pharmacological actions as an antioxidant, an anti-inflammatory, and an antibacterial (18). *In vivo* studies showed that bergenin administration to rats with 2,4,6-trinitrobenzenesulfonic acid (TNBS)-induced acute colitis significantly downregulated IFN-γ, NLRP3 inflammasome complex-forming proteins, IL-1β, and IL-18 in colonic tissue. Thereby blocking the canonical pathway of the NLRP3 inflammasome (53).

Since the pro-inflammatory macrophage phenotype plays a central role in intestinal mucosal barrier damage, there has been much interest in counteracting macrophage activation by dietary polyphenols to maintain intestinal barrier integrity (54, 55). A recent study showed that ligstroside aglycon (LA), an abundant phenolic compound in extra virgin olive oil (EVOO), inhibited canonical and non-canonical activation of NLRP3 inflammasome and modulated cyclo-oxygenase-2 (COX-2) in lipopolysaccharide (LPS)-stimulated murine peritoneal macrophages. This anti-inflammatory effect was associated with a significant antioxidant activity revealed by a decrease in NO production, inducible nitric oxide synthase (iNOS), and NADPH oxidase-1 (NOX-1) protein expression (56). Besides, rosmarinic acid nanovesicles protected the colonic mucosa from dextran sodium sulfate (DSS)-induced injury by regulating the NLRP3 inflammasome and restoring Nrf2/ heme oxygenase (HO-1) signaling pathway (52). It has also been demonstrated that hydroxytyrosol supplementation exhibits anti-inflammatory effects in murine ulcerative colitis models by promoting eubiosis. Moreover, hydroxytyrosol enhanced the colonic antioxidant capacity concomitant with NLRP3 inflammasome inhibition, revealed by caspase-1, ASC, IL-18, and IL-1β downregulation (22).

### 3.3. Liver disorders

Clinically, IBD and NAFLD frequently coexist (57). Liver diseases account for more than 45% of fatalities in developed countries and significantly contribute to worldwide morbidity and mortality since the liver is the main metabolically active and detoxification organ (58, 59). Numerous conditions, such as drug abuse, alcoholism, viral infections, metabolic abnormalities, and autoimmune reactions, can result in liver damage. Additionally, liver pathophysiological processes are frequently linked to inflammation and oxidative stress, suggesting a dual control of one another (58). Some hepatic pro-inflammatory cytokines, such as IL-1β, are rapidly produced after liver damage and directly activate hepatic stellate cells (HSCs), causing the conversion of these cells into myofibroblasts. The last secret a large amount of extracellular matrix (ECM) that leads to scar tissue formation. Many studies have associated increased pyroptosis with various illnesses, including liver abnormalities (58). Activation of NLRP3 inflammasome has emerged as the primary trigger of liver fibrosis and cirrhosis (58, 60). Moreover, caspase-1 and ASC are involved in the inflammatory and oxidative stress responses (61). The transcription factor Nrf2 orchestrates the cellular response to redox stress. Once activated, the cytosolic Nrf2 translocates to the nucleus, initiating gene expression of several antioxidant enzymes (62). Therefore, Nrf2 has become a main target for managing oxidative stress-associated disorders. Indeed, in addition to stimulating liver regeneration, Nrf2 has been reported to play intricate roles in the modulation of liver fibrosis, cancer, and inflammation (59). Likewise, NLRP3 upregulates hepatic Kelch-like ECH-related protein 1 (Keap-1), Nrf2 negative regulator, which may cause fibrogenesis as a result of ROS-induced pyroptosis (58). Hence, antioxidant therapy effectively counters ROS-mediated activation of the NLRP3 inflammasome in murine acute liver injury models (61). Another *in vivo* study demonstrated that chlorogenic acid, a hydroxycinnamic acid, upregulates the expression and the activation of Nrf2-related antioxidant genes, including HO-1, NAD(P)H:quinone oxidoreductase-1 (NQO1), and glutamate-cysteine ligase catalytic subunit (GCLC). Furthermore, this phenolic acid inhibited NLRP3 inflammasome activation revealed by caspase-1 and IL-1β proteins downregulation in Sprague–Dawley rats with carbon tetrachloride (CCl_4_)-induced acute liver injury (59).

Without excessive alcohol consumption, NAFLD is the most common cause of liver dysfunction in the Western world (63). NAFLD is not a single disorder. Instead, it refers to a variety of hepatic pathologies, from fatty liver (steatosis) to fatty liver with a pronounced inflammation and fibrosis (nonalcoholic steatohepatitis, or NASH) to cirrhosis and possibly hepatocellular cancer (64, 65). The NLRP3 inflammasome plays a crucial role in the development of NASH. Recent findings suggested that cannabidiol, a terpenophenolic compound, may diminish the risk for NASH by downregulating NF-κB and NLRP3 inflammasome signaling pathways in macrophages in high-fat high cholesterol (HFC) diet-fed mice (66).

Although the significant decrease in pathogen-caused foodborne diseases in developed countries, increased exposure to aflatoxin B1 (AFB1), produced by toxigenic *Aspergillus fungi*, has recently been found in some regions of the United States (67). Prolonged exposure to AFB1 has been suggested to induce hepatocyte pyroptosis and oxidative stress, which further results in liver injury (23). Importantly, curcumin mitigated AFB1-induced TNF-α, IL-6, and IL-1β pro-inflammatory cytokines production and ROS generation, resulting in curtailed necroptosis of chicken liver tissue (68). In line, another study showed that EGCG, a major flavonoid in green tea, inhibited NLRP3 inflammasome activation, which was associated with improved hepatic oxidative stress, cell apoptosis, necrosis, steatosis, and degeneration in CD-1 (ICR) mice (69). Emerging evidence from *in vivo* models highlights that the protective effects of curcumin against liver injuries and diseases are mainly exerted through Nrf-2 upregulation concomitant with NLRP3 and NF-κB pathways downregulation (20, 23, 70, 71). Data from a case–control study and a randomized controlled trial showed that anthocyanins, a flavonoid subclass, abrogate NLRP3 inflammasome, caspase-1, IL-1β, and IL-18 expression in subjects’ peripheral blood mononuclear cells (PBMCs) and plasma levels of IL-1β and IL-18 in patients with NAFLD (72). It has also been shown that cyanidin-3-O-β-glucoside, an anthocyanin, can attenuate alcoholic steatohepatitis through inducing NF-κB deacetylation and counteracting NLRP3 inflammasome activation (73).

The effects of various polyphenol classes on the studied inflammasome-related pathologies are summarized in Table 1.

**Table 1:** A summary of phenolic compounds’ regulation of the inflammasome pathway in pancreatitis, gastrointestinal, and liver disorders.

Inflammasome-associated disorders	Investigated dietary polyphenols			Study design and main findings		References
	Compounds used in the study	Class	Principal sources	Study details	Outcomes	
Pancreatitis	Rutin	Flavonoids	*Panicum virgatum*	Male albino *Wistar* rats treated with ethanol and cerulean, 5 weeks.	Downregulation of NLRP3-ASC-Caspase1 axis; Decreased IL-1β, IL-18, and TNF-α expression and production Reduced plasma levels of TBARS, Enhanced GPx, SOD, CAT activity	Aruna et al. (44) and Ramawat and Mérillon (74)
	Epigallocatechin-3-gallate	Flavonoids	Green tea carob flour	Exposure male Balb/C mice model with L-arginine induced acute pancreatitis, 4 weeks	Activation of SOD and GSH; Decline in MDA levels in the lung tissue; Counteraction of NLRP3 inflammasome activation.	Durazzo et al. (38) and Luo et al. (21)
	Apocynin	O-methyl catechols	Roots of *Apocynutit catinabinum*	Sodium taurocholate induced severe acute pancreatitis (SAP) in adult male *Wistar* rats, 7-8 weeks.	Low serum levels of TNF-α, IL-1β, and IL-6; Abolished NLRP3/NF-κB signaling cascade.	Jin et al. (45) and Stefanska and Pawliczak (75)
Gastrointestinal pathologies	Bergenin	Hydroxybenzoic acid derivatives	Plants of the genus *Peltophorum*	2,4,6-trinitrobenzenesulfonic acid (TNBS)-induced acute colitis in *Wistar* rats.	Blockade of canonical and non-canonical NLRP3/ASC inflammasome signaling pathways; Curtailment of pro-inflammatory proteins and cytokines: IL-1β, IL-10, IFN-ɣ, IL-1, IL-11, NF-κB, and STAT3.	Lopes de Oliveira et al. (53)
	Rosmarinic acid	Hydroxycinnamic acid	Cloves, cumin, fenugreek, parsley	dextran sodium sulphate (DSS)-induced acute colitis in C57BL/6 mice, 7 days.	Decrease in myeloperoxidase activity and TNF-α production; Downregulation of NLRP3, ASC, caspase-1, and IL-1β expression.	Marinho et al. (52) and Ramawat and Mérillon (74)
	Hydroxytyrosol	Secoiridoids	Olive oil	DSS-induced ulcerative colitis in Kunming male mice, 14 days.	Suppression of NLRP3, caspase-1, ASC, IL-18, and IL-1β expression; Maintenance of eubiosis.	Miao and Ph (76) and Ramawat and Mérillon (74)
Liver disorders	Thymol	Terpenophenolics	Thyme	BALB/c male mouse model with liver injury induced by lipopolysaccharides (LPS), 34 days.	Modulation of the expression of NLRP3, TNFα, IL-1β, and IL-18; Regulation of the apoptotic caspase-3 and − 9 gene expression and activation.	Dou et al. (77)
	Cannabidiol		Marijuana	C57BL/6 J male mice feeding with a high-fat high cholesterol (HFC) diet, 8 weeks.	Suppression of NF-κB and NLRP3 inflammasome activation.	Huang et al. (66)
	Chlorogenic acid	hydroxycinnamic acid	Coffee, tea, whole cereal grains, garlic, tomato	CCl_4_-induced acute liver injury in male Sprague–Dawley rats, 7 days.	Reduced expression of NLRP3, procaspase-1 and pro-IL-1β.	Shi et al. (59) and Nani et al. (18)
	Epigallocatechin-3-gallate	Flavonoids	Green tea carob flour	Mouse model of perfluorodecanoic acid (PFDA)-induced liver damage, 12 days.	Downregulation of hepatic NLRP3 signaling.	Durazzo et al. (38) and Wang et al. (69)
	Cyanidin-3-O-β-glucoside	Flavonoids	Deep-colored fruits and vegetables, flowers and fruits of *Elymus repens*, *Morus alba*, *Vitis vinifera*, *Vaccinium corymbosum* and *Vaccinium myrtillus*	Male C57BL/6 J mice fed with HFC plus ethanol high fat/high cholesterol diet, 7 days.	Suppression of NF-κB acetylation; Abrogation of NLRP3 inflammasome activation and pro-inflammatory cytokines release in hepatic cell lines.	Zhou et al. (73) and Cásedas et al. (78)
	Anthocyanins	Flavonoids	Raspberries, black cabbage, eggplant, radish, Strawberry	Patients with nonalcoholic fatty liver disease (NAFLD), 12 weeks.	Upregulation of NLRP3, caspase-1, IL-1β, and IL-18 mRNAexpression in peripheral blood mononuclear cells (PBMCs).	Nani et al. (18) and Zhu et al. (72)

## 4. Conclusion

Western lifestyle characterized by nutritional transition is a major risk factor for inflammasome-associated inflammation and illnesses. Nutritional approaches encourage polyphenol-rich foods as a prominent strategy for NLRP3 inflammasome-related disorders prevention and management. The current review sheds light on how dietary polyphenols can regulate NLRP3 inflammasome activation in pancreatitis, gastrointestinal and liver diseases. Overall, polyphenols counteract inflammasome complex assembly, downregulating its downstream substrates, IL-1β and IL-18. Moreover, polyphenols may modulate oxidative stress associated with inflammation in these NLRP3 inflammasome-linked pathologies via upregulating Nrf-2 and abolishing NF-κB pathway.

## Author contributions

AN conceptualized the topic, researched and analyzed the literature and wrote the manuscript, and revised the manuscript critically. WT researched and analyzed the literature, wrote the manuscript, and constructed the figures. All authors contributed to the article and approved the submitted version.

## Funding

The author(s) received no financial support for this article’s research, authorship, and/or publication.

## Conflict of interest

The authors declare that the research was conducted in the absence of any commercial or financial relationships that could be construed as a potential conflict of interest.

## Publisher’s note

All claims expressed in this article are solely those of the authors and do not necessarily represent those of their affiliated organizations, or those of the publisher, the editors and the reviewers. Any product that may be evaluated in this article, or claim that may be made by its manufacturer, is not guaranteed or endorsed by the publisher.

## Supplementary material

The Supplementary material for this article can be found online at: https://www.frontiersin.org/articles/10.3389/fnut.2023.1157572/full#supplementary-material
