# Supplementary figures and images for: Targeting inflammasome pathway by polyphenols as a strategy for pancreatitis, gastrointestinal and liver diseases management: an updated review
Source: Front Nutr. 2023 Aug 31;10:1157572. doi: 10.3389/fnut.2023.1157572 (PMC10513047; doi:10.3389/fnut.2023.1157572)

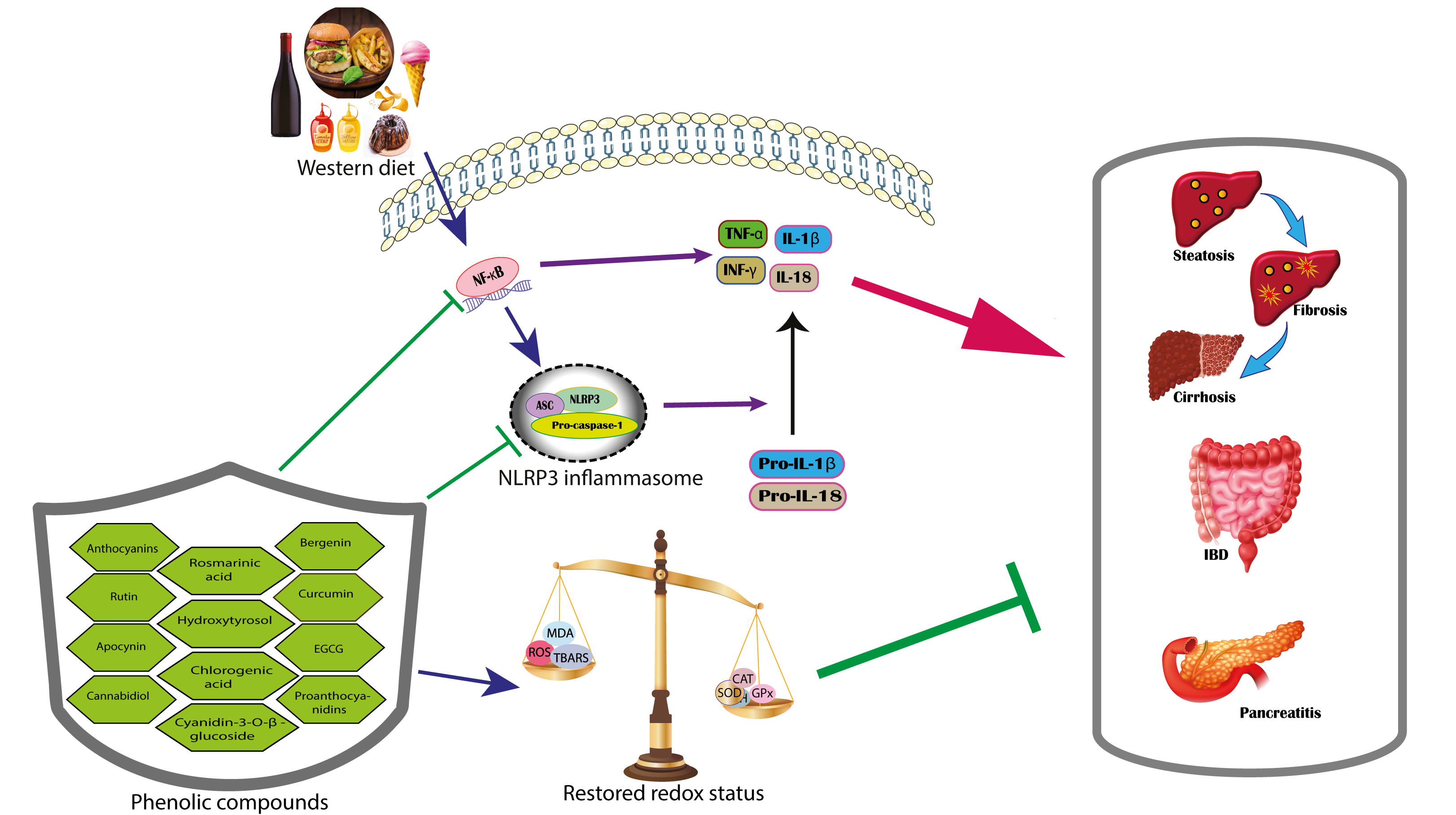

Supplement: Supplementary file 1 [file Image_1.TIF]
